# Bruton Tyrosine Kinase Inhibitors in Mantle Cell Lymphoma: What Are the Current Options?

**DOI:** 10.1111/ejh.70036

**Published:** 2025-09-13

**Authors:** Santino Caserta, Enrica Antonia Martino, Ernesto Vigna, Antonella Bruzzese, Nicola Amodio, Eugenio Lucia, Virginia Olivito, Caterina Labanca, Francesco Mendicino, Fortunato Morabito, Massimo Gentile

**Affiliations:** ^1^ Hematology Unit, Department of Onco‐Hematology AO of Cosenza Cosenza Italy; ^2^ Department of Experimental and Clinical Medicine University of Catanzaro Catanzaro Italy; ^3^ AIL Sezione di Cosenza Cosenza Italy; ^4^ Department of Pharmacy Health and Nutritional Science, University of Calabria Rende Italy

**Keywords:** BTKi, MCL, therapy

## Abstract

Mantle Cell Lymphoma (MCL) is an aggressive B‐cell non‐Hodgkin lymphoma characterized by the hallmark t(11;14)(q13;q32) translocation, resulting in cyclin D1 overexpression. Predominantly affecting elderly males, MCL exhibits marked clinical and biological heterogeneity, ranging from indolent SOX11‐negative variants to highly proliferative blastoid variants. Despite initial responsiveness to chemoimmunotherapy, relapse is frequent, and median overall survival remains limited. Aberrant B‐cell receptor (BCR) signaling—mediated by kinases including Bruton's tyrosine kinase (BTK)—plays a central role in MCL pathogenesis. BTK inhibitors (BTKis) such as ibrutinib (as monotherapy or combined with R‐CHOP/R‐DHAP), acalabrutinib (with rituximab and bendamustine), and pirtobrutinib have significantly improved outcomes of relapsed/refractory disease. However, their efficacy is challenged by resistance mutations (e.g., BTK C481S) and off‐target toxicities. Next‐generation reversible BTKis represent an important advance, offering activity in the setting of resistance and improved tolerability. Resistance is further sustained by the tumor microenvironment through stromal support and immunosuppressive cellular interactions. Consequently, combination strategies incorporating BTKis with BCL‐2 inhibitors, monoclonal antibodies, and cellular therapies are being investigated to enhance response depth and durability. In parallel, biomarker‐driven approaches and precision‐medicine strategies are emerging to personalize disease monitoring and treatment selection. Collectively, these developments underscore the evolving role of BTK inhibition within broader immune‐based and targeted treatment paradigms in MCL.

## 1 Introduction

### 1.1 Overview of Mantle Cell Lymphoma

Mantle cell lymphoma (MCL) is a rare and aggressive subtype of non‐Hodgkin lymphoma (NHL), accounting for ~6% to 8% of all NHL cases [1]. It primarily affects older adults, with a median age at diagnosis of around 65 years, and exhibits a marked male predominance, with a male‐to‐female ratio of ~3:1. MCL arises from naïve B‐cells located in the mantle zone of lymphoid follicles. Its hallmark genetic feature is the chromosomal translocation t(11;14)(q13;q32), which leads to overexpression of the CCND1 gene encoding cyclin D1, thereby promoting dysregulated cell cycle progression and uncontrolled cellular proliferation [2, 3, 4].

The pathogenesis of MCL involves multiple molecular abnormalities beyond cyclin D1 overexpression. These include inactivation of tumor suppressor genes *TP53* and *ATM*, constitutive activation of the B‐cell receptor (BCR) signaling pathway, and defects in the DNA damage response—factors that collectively drive genomic instability and resistance to apoptosis. Molecular profiling has enabled the identification of biologically distinct MCL subtypes, such as the indolent SOX11‐negative variant and the more aggressive blastoid and pleomorphic variants, the latter being associated with poorer clinical outcomes [5]. The integration of high‐throughput technologies—such as cell sorting, exome, and transcriptome sequencing—has enabled precise molecular characterization of malignant B cells in MCL. These approaches continue to uncover novel somatic mutations, gene expression signatures, and therapeutic vulnerabilities that support the implementation of personalized treatment strategies [6].

Clinically, MCL typically presents at an advanced stage (stage III/IV) with generalized lymphadenopathy, splenomegaly, bone marrow infiltration, and frequent extranodal involvement, particularly in the gastrointestinal tract. Although the disease often responds initially to chemoimmunotherapy, it is characterized by a high relapse rate and eventual development of treatment resistance, resulting in a median overall survival (OS) of ~5 to 7 years. Disease behavior can range from indolent forms requiring observation to rapidly progressive variants [7].

Prognostic tools such as the mantle cell lymphoma international prognostic index (MIPI) and biomarkers like Ki‐67 proliferation index are widely used to stratify patients. In detail, the MIPI score has been widely validated and is based on clinical parameters such as age, performance status, lactate dehydrogenase (LDH) levels, and leukocyte count. However, despite its robust prognostic value, it is not routinely applied to guide therapeutic decisions in many centers, where treatment selection is primarily driven by patients’ fitness, comorbidities, and disease biology. In recent years, advances in targeted therapies, including Bruton's tyrosine kinase inhibitors (BTKis), BCL‐2 inhibitors, and chimeric antigen receptor (CAR) T cell therapy, have significantly improved outcomes in relapsed/refractory (R/R) MCL. However, the disease remains incurable in the majority of cases, underscoring the persistent need for innovative treatment strategies, ongoing clinical research, and deeper biological insights [8, 9].

#### 1.1.1 Rationale for Targeting B‐Cell Receptor Signaling

Targeting B‐cell receptor (BCR) signaling has emerged as a highly effective therapeutic strategy across various B‐cell malignancies, including MCL. This approach is based on the pivotal role of BCR signaling in normal B‐cell development and its pathological activation in malignant B cells. In neoplastic contexts, BCR signaling can become constitutively active or antigen‐independent, delivering continuous survival and proliferative signals that support tumor growth, facilitate immune evasion, and contribute to resistance to conventional chemotherapies [10].

The BCR signaling cascade involves a network of kinases, including Lyn, spleen tyrosine kinase (SYK), BTK, and phosphoinositide 3‐kinase (PI3K). These kinases orchestrate the activation of downstream pathways such as NF‐κB, MAPK, and PI3K‐AKT–mTOR, which regulate cellular proliferation, survival, metabolism, and trafficking. Inhibiting key components of this cascade disrupts the oncogenic signaling and impairs the interaction between malignant B‐cell clones and their supportive microenvironment. BTKis such as ibrutinib and acalabrutinib, along with newer reversible agents like pirtobrutinib, have demonstrated significant clinical efficacy, particularly in R/R MCL [11].

In addition to inducing apoptosis and reducing tumor cell viability, BCR pathway inhibitors also impair chemokine‐mediated migration and adhesion of tumor cells within protective lymphoid niches. This microenvironmental interference further enhances therapeutic responses. However, the emergence of resistance remains a major clinical challenge, emphasizing the need for rational combination strategies and the development of next‐generation inhibitors [12].

### 1.2 Role of BTK in BCR Signaling

BCR is a central mediator of the BCR signaling pathway, which is essential for normal B‐cell development and immune function. Aberrant activation of this pathway contributes to lymphomagenesis, making BTK a key therapeutic target in several B‐cell lymphomas, including MCL [13].

Ibrutinib, the first FDA‐approved covalent BTKi, demonstrated significant clinical efficacy in treating R/R MCL, chronic lymphocytic leukemia, and other B‐cell malignancies. Second‐generation covalent inhibitors, such as acalabrutinib and zanubrutinib, were developed to improve kinase selectivity, reduce off‐target effects, and enhance tolerability. However, resistance to covalent BTKis, most commonly mediated by mutations at the C481 binding site, remains a clinical challenge. This has led to the development of non‐covalent BTK inhibitors like pirtobrutinib, which retain activity despite C481 mutations [14].

Nonetheless, resistance has also emerged to non‐covalent inhibitors, including novel kinase‐deficient BTK mutations. These findings suggest that BTK's role extends beyond its catalytic activity to include scaffolding functions that support oncogenic signaling. Consequently, new therapeutic strategies aim to target both the enzymatic and non‐enzymatic functions of BTK. Among these, proteolysis‐targeting chimeras (PROTACs) such as NX‐2127, NX‐5948, and BGB‐16673 represent a novel class of therapeutics that induce the selective degradation of BTK and other oncogenic proteins like IKZF1 and IKZF1/3, potentially overcoming resistance mechanisms seen with traditional inhibitors [15].

Unlike conventional inhibitors, which rely on continuous occupancy of the BTK active site, PROTACs act catalytically, allowing a single molecule to trigger the degradation of multiple BTK molecules. This distinct pharmacologic mechanism may offer more sustained efficacy and a reduced likelihood of resistance. Overall, targeting both the catalytic and the scaffold functions of BTK represents a promising approach to improve outcomes in B‐cell malignancies, particularly in R/R settings, and may broaden therapeutic options across diverse lymphoma subtypes [16].

## 2 BTK Inhibitors

### 2.1 BTK as a Target in B‐Cell Malignancies

BTKis are broadly classified into two main categories based on their binding mechanism: irreversible (covalent) and reversible (non‐covalent) inhibitors. These classes differ significantly in their pharmacological properties, resistance mechanisms, and clinical applications, particularly in the management of B‐cell malignancies [17, 18, 19, 20].

Irreversible BTKis—such as including ibrutinib, acalabrutinib, and zanubrutinib—form a covalent bond with the cysteine residue (C481) in the ATP‐binding site of BTK. This bond leads to the permanent inactivation of the enzyme until new BTK proteins are synthesized. Covalent binding results in sustained inhibition of BCR signaling pathways even after the drug has been cleared from circulation, allowing for once‐ or twice‐daily dosing. These agents have shown high clinical efficacy in MCL and other B‐cell malignancies. However, off‐target effects are a concern, particularly with ibrutinib, which also inhibits other kinases such as EGFR, ITK, and TEC. These off‐target interactions are associated with adverse events, including atrial fibrillation, bleeding, and hypertension [21].

**FIGURE 1:**
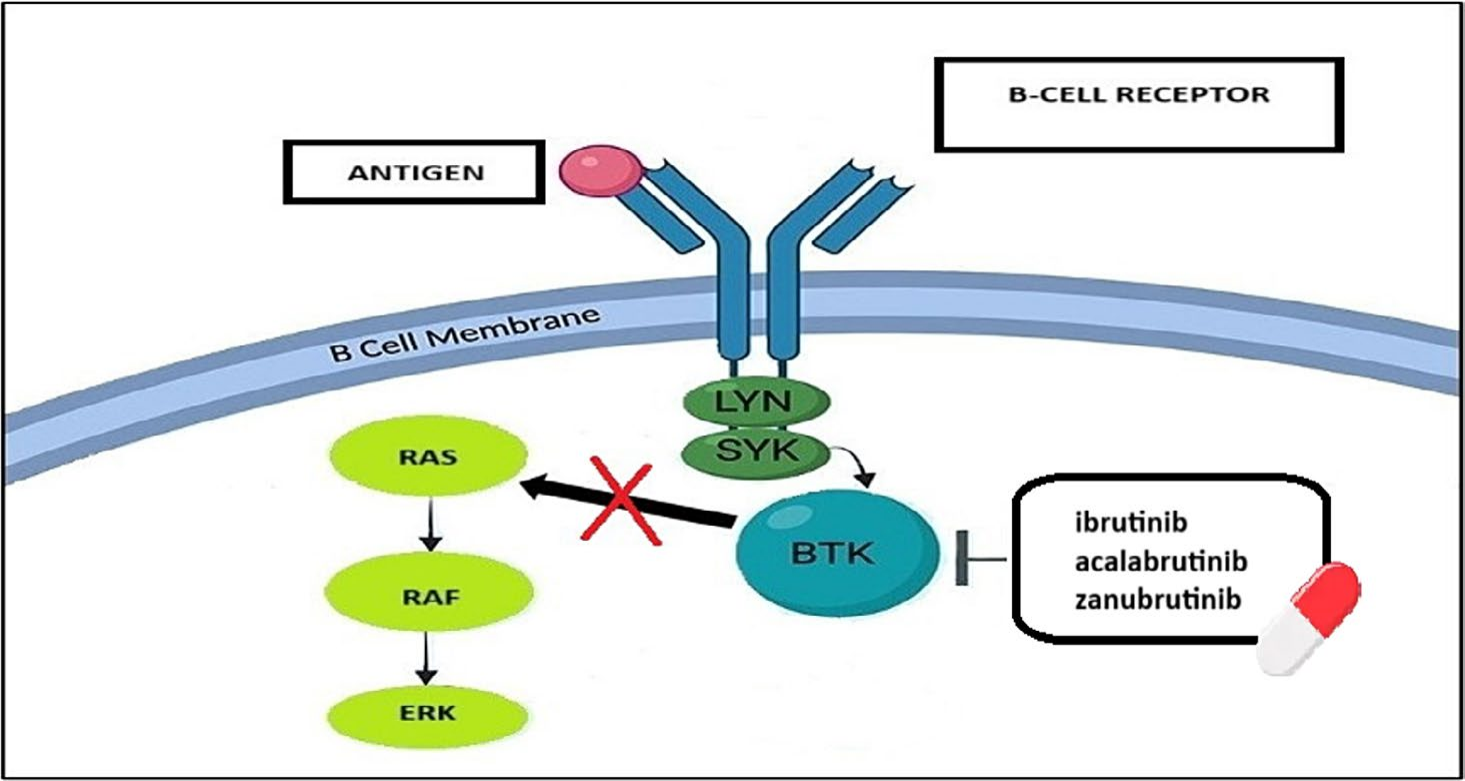
Ibrutinib, acalabrutinib, and zanubrutinib selectively inhibits BTK, promoting apoptosis and blocking proliferation of malignant B cells.

Reversible BTKis, such as pirtobrutinib and nemtabrutinib, bind non‐covalently to BTK—typically at sites distinct from C481—enabling inhibition of both wild‐type and C481‐mutated BTK. This is particularly relevant for patients who acquire resistance to covalent inhibitors due to mutations at the C481S residue (e.g., C481S or C481R), which prevent effective covalent binding. Reversible inhibitors offer a broader therapeutic window in R/R settings and tend to exhibit greater kinase selectivity, potentially minimizing toxicity. Although their non‐covalent binding requires sustained plasma concentrations for continuous BTK inhibition, recent advances in drug design have yielded favorable pharmacokinetic profiles that support once‐daily dosing [22] (Figure 1).

Drug availability for MCL differs between regions. In the United States (US), ibrutinib, acalabrutinib, and zanubrutinib are approved by the Food and Drug Administration (FDA) for use primarily in the relapsed/refractory setting, and pirtobrutinib has received accelerated approval for patients previously treated with at least two prior lines of therapy, including a covalent BTKi. In Europe, the European Medicines Agency (EMA) has approved ibrutinib and zanubrutinib for relapsed/refractory MCL, while acalabrutinib is authorized for other B‐cell malignancies but not currently for MCL. Pirtobrutinib remains investigational in Europe pending EMA evaluation. These regional differences need to be considered when defining treatment strategies alongside patient characteristics such as age, comorbidities, and disease biology.

### 2.2 Pharmacodynamics/Pharmacokinetics of BTKis

The pharmacodynamic (PD) and pharmacokinetic (PK) properties of BTKis play a critical role in determining their efficacy, safety, and clinical utility in B‐cell malignancies.

Pharmacodynamically, BTKis exert their antitumor activity by inhibiting BTK‐mediated signaling pathways—such as NF‐κB, AKT, and MAPK—thereby disrupting key processes including malignant B‐cell proliferation, survival, adhesion, and migration. Irreversible BTKis (e.g., ibrutinib, acalabrutinib, and zanubrutinib) covalently bind to the C481 residue within the ATP‐binding site of BTK, leading to sustained inhibition even after plasma concentrations decline. This results in a prolonged pharmacodynamic effect and supports once‐ or twice‐daily dosing, depending on the specific agent. In contrast, non‐covalent BTKis (e.g., pirtobrutinib) bind reversibly and independently of the C481 residue, retaining efficacy in the presence of resistance mutations like C481S. These agents offer more flexible PD profiles and the potential to overcome acquired resistance [23].

Pharmacokinetically, BTKis display distinct absorption, distribution, metabolism, and excretion characteristics. Ibrutinib is rapidly absorbed after oral administration, with peak plasma concentrations reached within 1 to 2 h and a short half‐life (~4–6 h). Despite its short plasma half‐life, its covalent mechanism ensures prolonged BTK inhibition. Ibrutinib is extensively metabolized by cytochrome P450 3A4 (CYP3A4), raising the potential for drug–drug interactions. Acalabrutinib shares a similar metabolic profile but is more selective, contributing to a more favorable off‐target effect profile. Zanubrutinib demonstrates higher bioavailability and deeper BTK occupancy in lymphoid tissues, enhancing its therapeutic activity. Pirtobrutinib, a reversible non‐covalent inhibitor, has a longer half‐life (~19–21 h), allowing for sustained BTK inhibition and once‐daily dosing even in the context of resistance [24].

The PD/PK characteristics of BTKis inform critical aspects of clinical use, including dosing strategies, toxicity management, and combination regimens. Drug selectivity and metabolic pathways influence tolerability and safety, while bioavailability and tissue distribution are key determinants of clinical efficacy.

### 2.3 Resistance Mechanisms

Resistance to BTKis in MCL arises from a complex interplay of intrinsic and extrinsic mechanisms, with the tumor microenvironment (TME) playing a central role. While genetic alterations and clonal evolution contribute to resistance, accumulating evidence indicates that the protective influence of the TME is indispensable for malignant cells' survival under therapeutic pressure.

In contrast to chronic lymphocytic leukemia (CLL) and Waldenström macroglobulinemia (WM), where *BTK* C481 mutations are common and represent the dominant cause of resistance, such mutations are rare in MCL. Instead, alternative mechanisms—including mutations in *PLCG2*, constitutive NF‐κB activation, and alterations in cell cycle regulators like *CCND1* and *TP53*—have been described. However, these changes alone do not fully explain the widespread resistance observed clinically. Rather, they act in concert with signals derived from the TME, which enhance tumor fitness and promote therapeutic escape [25].

The TME functions as a sanctuary for malignant B cells, orchestrating a multifaceted resistance network. Stromal cells play a particularly central role by secreting chemokines such as CXCL12 and CXCL13 and expressing adhesion molecules including VLA‐4 and CXCR4, thereby activating both BTK‐dependent and BTK‐independent pathways. These interactions restore the activity of critical effectors, including NF‐κB, PI3K/AKT, and MAPK, supporting proliferation and survival despite BTK blockade. Nurse‐like cells further sustain minimal residual disease through strong anti‐apoptotic signals, while tumor‐associated macrophages (TAMs) and myeloid‐derived suppressor cells (MDSCs) establish an immunosuppressive niche. By releasing cytokines such as IL‐10 and TGF‐β, these immune subsets suppress cytotoxic T‐cell function and facilitate immune evasion, creating a microenvironment favorable to resistant clones. Importantly, some myeloid subsets also express BTK, underscoring the complexity of the microenvironmental contributions to drug resistance [26].

To better summarize these multiple layers of resistance, Table 1 highlights the key mechanisms by which malignant B cells evade BTKi therapy. This table summarizes the key, overlapping mechanisms by which malignant B cells evade BTKi therapy, highlighting pathways that should be considered when designing combination strategies targeting both intrinsic and extrinsic resistance drivers.

**TABLE 1:** Mechanisms of resistance in BTK inhibitors.

Mechanism	Description	Key components	Impact on BTKi resistance
Protective cellular niches	Specialized TME cells shield malignant B cells from apoptosis despite BTK inhibition	Nurse‐like cells (NLCs), stromal cells	Sustain minimal residual disease, limiting the complete eradication of leukemic cells
Paracrine signaling by BTK‐mutant clones	Mutant subclones secrete cytokines that promote proliferation and survival of neighboring wild‐type B cells	Cytokines (various)	Promote tumor persistence and clonal evolution despite therapy
Chemokine‐mediated survival signals	Stromal secretion of chemokines activates BTK‐dependent and independent pathways enhancing malignant cell survival	CXCL12, CXCL13, CXCR4, VLA‐4	Reactivates downstream signaling despite BTK inhibition, maintaining proliferation and drug resistance
Immunosuppressive microenvironment	Immune cells create a niche that impairs anti‐tumor immunity via immunosuppressive cytokines	Tumor‐associated macrophages, MDSCs, IL‐10, TGF‐β	Suppresses cytotoxic T‐cell function, facilitating immune evasion and allowing drug‐resistant clones to thrive
Structural protection by stromal networks	Physical interactions with stromal cells support malignant cell survival and protect from therapeutic effects	Adhesion molecules (VLA‐4, CXCR4)	Provides survival advantage and physical niche resistant to BTK inhibition

Although cell‐intrinsic mechanisms such as clonal evolution and activation of alternative signaling cascades—including PI3K/AKT/mTOR and MAPK—play a complementary role, the persistence of malignant cells in MCL remains highly dependent on the continuous support of the TME. This microenvironment not only provides structural protection and soluble survival factors but also establishes dynamic feedback loops that reinforce resistance mechanisms at multiple levels.

In summary, the TME is not a passive bystander but a fundamental driver of resistance to BTKis in MCL. Its influence extends beyond supportive interactions, actively reprogramming signaling and immune landscapes to promote malignant cell persistence. Consequently, therapeutic strategies that target both intrinsic genetic mechanisms and the protective TME—such as combining BTKis with CXCR4 antagonists, PI3K inhibitors, or immune modulators—are likely required to achieve deeper and more durable remissions.

### 2.4 Safety, Tolerability, and Management of Adverse Events

The long‐term use of BTKis is often limited by adverse events (AEs), which vary in frequency and severity depending on the agent's kinase selectivity, patient comorbidities, and prior treatments. First‐generation BTKis such as ibrutinib are associated with a broader kinase inhibition profile, contributing to a higher incidence of cardiovascular and bleeding complications. Atrial fibrillation, hypertension, and bleeding are among the most common AEs, largely due to ibrutinib's off‐target inhibition of kinases such as EGFR, ITK, and TEC. Other frequently reported AEs include gastrointestinal symptoms (e.g., diarrhea, nausea), rash, and arthralgia [27].

Second‐generation covalent BTKis (e.g., acalabrutinib, zanubrutinib) were designed to enhance selectivity for BTK and reduce off‐target toxicities. Head‐to‐head trials have demonstrated lower rates of atrial fibrillation and bleeding events with these agents compared to ibrutinib. Nonetheless, AEs still occur and warrant careful monitoring and supportive management. Headache is a notable side effect of acalabrutinib, typically transient and responsive to analgesics. Cytopenias and infections—especially in heavily pretreated patients—remain relevant safety concerns with all BTKis, necessitating regular blood count monitoring and prophylactic strategies in high‐risk individuals.

Reversible (non‐covalent) BTKis, such as pirtobrutinib, exhibit favorable safety profiles even in patients with prior BTKi exposure or resistance. Their reduced cardiac and bleeding toxicity is likely attributable to minimal off‐target inhibition, making them appealing for patients intolerant to earlier‐generation agents.

These toxicities are of particular concern in elderly patients, who constitute the majority of MCL cases and are more susceptible to cardiovascular events and drug discontinuation. By contrast, younger, transplant‐eligible patients generally tolerate covalent BTKis more effectively, particularly when administered in combination with intensive chemoimmunotherapy.

The choice of BTKis in frail patients with MCL and pre‐existing cardiac comorbidities remains challenging, as cardiovascular safety is a critical consideration. Ibrutinib has been associated with atrial fibrillation, hypertension, and bleeding complications—adverse events that are especially problematic in elderly or comorbid patients. In this context, second‐generation covalent BTKis such as acalabrutinib and zanubrutinib are generally preferred. Both agents display improved kinase selectivity and reduced off‐target activity, resulting in lower rates of cardiovascular toxicity. Zanubrutinib, in particular, is associated with a very low incidence of atrial fibrillation and hypertension, making it a suitable option for patients with elevated cardiovascular risk. Pirtobrutinib has also demonstrated a favorable cardiovascular safety profile and represents a promising alternative once it becomes available outside clinical trials [28].

Effective management of BTKi‐related AEs requires early identification, dose adjustments, and supportive care. For atrial fibrillation, a careful balance between rate/rhythm control and anticoagulation is essential, given the associated bleeding risk. In cases of persistent toxicity, switching to a more selective BTKi or discontinuing therapy in favor of alternative agents may be necessary.

## 3 BTKi In MCL

### 3.1 Ibrutinib Plus R‐CHOP/R‐DHAP as First‐Line Therapy

Although MCL often responds to initial chemoimmunotherapy, relapse is common. The demonstrated efficacy of ibrutinib in the R/R setting has prompted its investigation as a component of frontline regimens.

The phase III TRIANGLE trial evaluated the addition of ibrutinib to standard induction therapy—R‐CHOP alternating with R‐DHAP—with or without ASCT in previously untreated MCL patients ≤ 65 years. This study represented a significant step in integrating targeted therapy into frontline treatment protocols. The rationale for adding ibrutinib was to enhance the depth of response, prolong remission duration, and potentially reduce the need for transplantation [29].

In the ibrutinib‐containing arms, the overall response rate (ORR) exceeded 90%, with complete response (CR) rates reaching up to 80%. Notably, patients who received ibrutinib without ASCT demonstrated progression‐free survival (PFS) outcomes that were non‐inferior to those who underwent transplant, with median PFS not reached at the time of early follow‐up. Preliminary data also suggest a favorable trend in overall survival (OS), though long‐term results are awaited.

The addition of ibrutinib was associated with increased hematologic toxicity, including Grades 3and 4 neutropenia and thrombocytopenia, as well as a higher incidence of febrile neutropenia and infections, especially during induction. Non‐hematologic AEs included diarrhea, hypertension, and atrial fibrillation (reported in ~5%–8% of patients). However, these toxicities were generally manageable through supportive care, dose modifications, and close monitoring.

The TRIANGLE trial supports the incorporation of ibrutinib into first‐line regimens for MCL, showing that it can deepen remission and may allow for omission of ASCT in selected patients—potentially redefining the standard of care [30].

In summary, the combination of R‐CHOP/R‐DHAP as frontline therapy demonstrates high efficacy and an acceptable safety profile in MCL, offering a promising approach to reduce transplant reliance and improve long‐term disease control.

### 3.2 Ibrutinib Single Agent for Second‐Line Therapy

Ibrutinib has demonstrated robust activity as a single agent in R/R MCL [31].

By selectively inhibiting BTK—a key component of the B‐cell receptor signaling pathway—it disrupts downstream survival signals, promotes apoptosis, and impairs proliferation of malignant B cells. In the pivotal phase II PCYC‐1104 trial, ibrutinib achieved an ORR of 68%, including a CR rate of 21%. The median PFS was 13.9 months, and the median OS was 22.5 months. Extended follow‐up confirmed that a subset of patients can achieve durable remissions [32].

The toxicity profile of ibrutinib is generally favorable compared to conventional chemotherapy. The most commonly reported AEs include diarrhea (50%), fatigue (41%), nausea (31%), and peripheral edema (25%). Clinically significant bleeding events and atrial fibrillation occur in approximately 5% to 10% of patients and require careful monitoring and management. Grades 3 and 4 neutropenia and thrombocytopenia are relatively uncommon, and with appropriate prophylaxis, the risk of opportunistic infections remains moderate.

Given its oral administration, manageable toxicity, and feasibility in the outpatient setting, ibrutinib is particularly well‐suited for older patients or those ineligible for high‐dose chemotherapy or ASCT. However, acquired resistance—often due to mutations in BTK (e.g., C481S) or downstream effectors such as PLCγ2—can limit long‐term efficacy, underscoring the need for sequential therapies or combination strategies.

In conclusion, ibrutinib monotherapy represents an effective and well‐tolerated second‐line option in MCL, offering substantial clinical benefit and improved quality‐of‐life advantages, particularly in patients with limited therapeutic alternatives.

### 3.3 Acalabrutinib Plus Rituximab and Bendamustine as First‐Line Therapy

Acalabrutinib is a second‐generation BTK inhibitor distinguished by its enhanced selectivity for BTK compared to first‐generation agents such as ibrutinib. This improved specificity minimizes off‐target kinase inhibition, thereby lowering the incidence of associated toxicities—an important consideration in the treatment of MCL [33]. The combination of acalabrutinib with rituximab and bendamustine (the BR regimen) has been evaluated as a first‐line therapy in treatment‐naïve MCL patients, demonstrating encouraging efficacy alongside a manageable safety profile.

This triplet regimen leverages the complementary mechanisms of action of each agent: bendamustine exerts direct cytotoxic effects on malignant cells; rituximab mediates antibody‐dependent cellular cytotoxicity via CD20 targeting; and acalabrutinib disrupts BTK‐driven survival and proliferation signaling in MCL cells. Together, these agents enhance anti‐lymphoma activity. Phase II clinical trials have reported high ORR and CR rates, with deeper responses observed compared to BR alone [34].

Regarding safety, the addition of acalabrutinib does not substantially increase hematologic toxicity beyond that associated with BR. The most commonly reported AEs include neutropenia, thrombocytopenia, and anemia, consistent with the expected profile of bendamustine. Non‐hematologic toxicities are generally mild to moderate and include headache, diarrhea, and fatigue. Importantly, due to its selective inhibition profile, acalabrutinib is associated with a lower incidence of atrial fibrillation and bleeding events compared to ibrutinib, improving treatment tolerability, particularly in elderly patients or those with comorbidities [35].

In summary, the combination of acalabrutinib, rituximab, and bendamustine represents a promising first‐line treatment option in MCL, effectively balancing potent efficacy with an acceptable safety profile. Ongoing phase III trials are expected to further define its role in improving long‐term outcomes and quality of life in this challenging disease.

### 3.4 Pirtobrutinib Single‐Agent as Third‐Line Therapy

**FIGURE 2:**
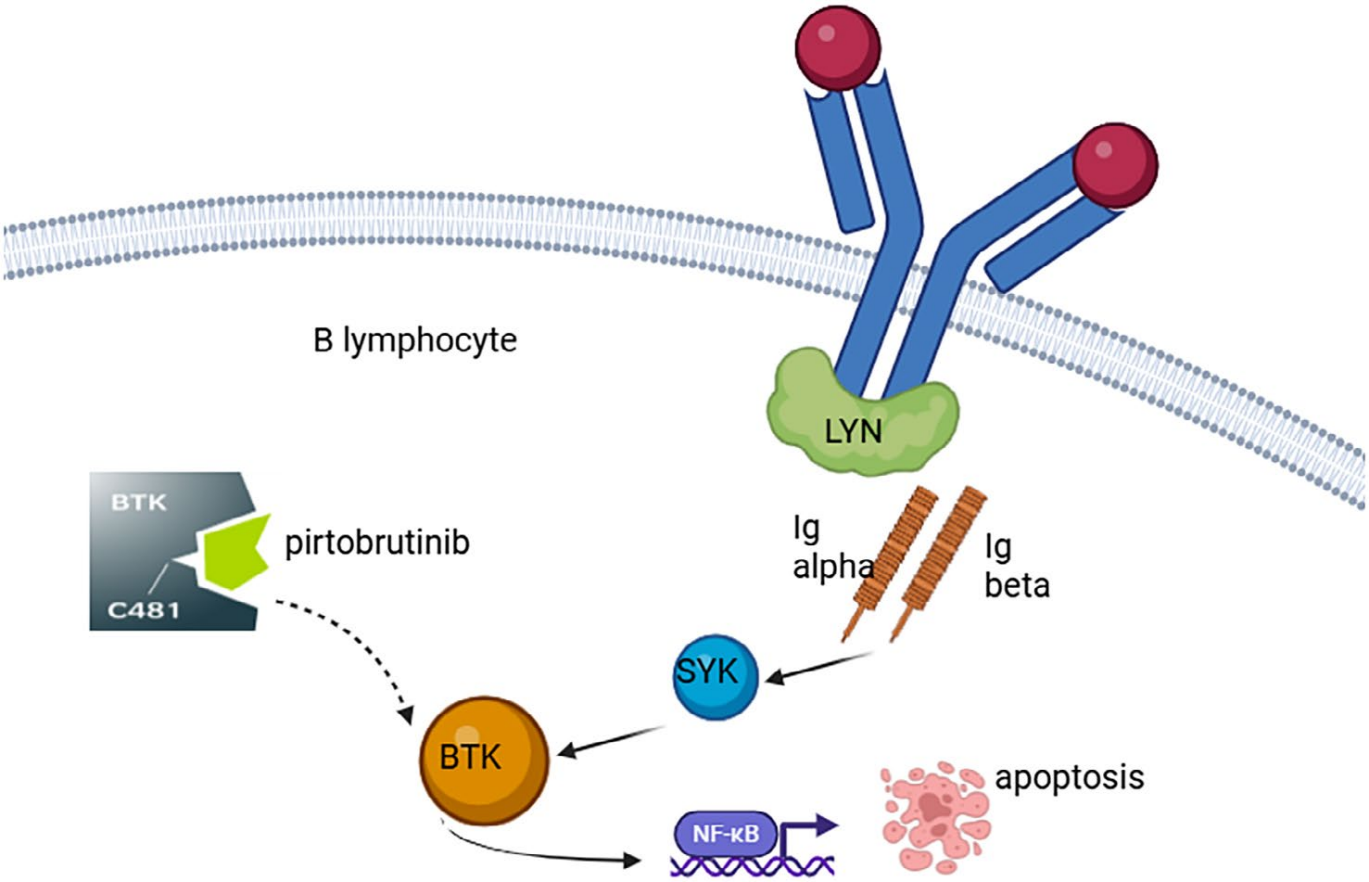
Pirtobrutinib inhibits BTK through a reversible binding that is independent of the cysteine residue, even in the presence of mutations at the C481 site. Created in BioRender.com.

Pirtobrutinib is a highly selective, non‐covalent (reversible) BTK inhibitor that has garnered significant interest in the treatment of B‐cell malignancies, particularly MCL, where resistance to covalent BTK inhibitors poses a major clinical challenge. Unlike first‐generation covalent BTKis, which irreversibly bind to the cysteine‐481 (C481) residue within the BTK active site, pirtobrutinib binds reversibly and does not rely on interaction with this residue. This distinct mechanism of action enables pirtobrutinib to retain activity in the presence of C481 mutations, a common resistance mechanism observed in patients who relapse following covalent BTKis (Figure 2).

Clinical evaluation of pirtobrutinib in the phase I/II BRUIN trial has demonstrated robust antitumor activity as a monotherapy in heavily pretreated patients with R/R MCL. The ORR in this challenging patient population was notable, with a substantial proportion of patients achieving CR or PR, highlighting the drug's potential to induce durable remissions even after prior exposure to covalent BTKi. These findings underscore the clinical relevance of pirtobrutinib's distinct mechanism of action and support its potential to redefine treatment strategies in managing resistant MCL [36].

The most commonly reported AEs are typically low grade (Grade 1 or 2) and include fatigue, diarrhea, contusion, and cough. Hematologic toxicities such as anemia, neutropenia, and thrombocytopenia have been observed but are less frequent and generally manageable with supportive measures, including dose adjustments. Notably, pirtobrutinib is associated with a markedly reduced incidence of cardiovascular toxicities, such as atrial fibrillation and hypertension, which have limited the long‐term use of earlier BTKis. Bleeding complications remain rare and predominantly mild in severity.

Serious AEs are infrequent, and treatment discontinuations due to toxicity are uncommon, further supporting the agent's favorable tolerability profile. Collectively, these clinical data establish pirtobrutinib as a promising therapeutic option for patients with R/R MCL, particularly those who have exhausted multiple prior therapies, including covalent BTKis. Ongoing phase III clinical trials are expected to validate these results and further clarify pirtobrutinib's role within the evolving treatment landscape of MCL [37]. Table 2 provides a comprehensive overview of the BTKis discussed, summarizing their pharmacologic class, binding mechanism, approved indications, key efficacy outcomes, and notable safety considerations in MCL.

**TABLE 2:** Comparative overview of BTK inhibitor–based regimens in mantle cell lymphoma: mechanisms, efficacy, and toxicity profiles.

Regimen	BTKi class	Mechanism of action	Clinical setting	Clinical efficacy	Hematological toxicities	Non‐hematological toxicities
Ibrutinib + R‐CHOP/R‐DHAP	First‐generation covalent BTKi	Ibrutinib: selective BTK inhibition; R‐CHOP/R‐DHAP: cytotoxic and anti‐CD20 activity	First‐line (intensive therapy)	ORR > 90%; CR ~80%; median PFS not reached (early follow‐up)	Neutropenia, thrombocytopenia; febrile neutropenia; infections	Diarrhea, hypertension, atrial fibrillation
Ibrutinib monotherapy	First‐generation covalent BTKi	Selective BTK inhibition	Relapsed/refractory (second‐line)	ORR 68%; CR 21%; median PFS 13.9 months	Neutropenia, thrombocytopenia	Diarrhea, fatigue, nausea, peripheral edema, bleeding, atrial fibrillation
Acalabrutinib + rituximab + bendamustine	Second‐generation covalent BTKi	Acalabrutinib: selective BTK inhibition; rituximab: anti‐CD20 cytotoxicity; Bendamustine: cytotoxicity	First‐line (chemoimmunotherapy)	High ORR and CR; deeper responses versus BR alone	Neutropenia, thrombocytopenia, anemia	Headache, diarrhea, fatigue
Pirtobrutinib monotherapy	Highly selective, non‐covalent (reversible) BTKi	Reversible BTK inhibition independent of C481 residue	Relapsed/refractory (third‐line, post‐BTKi failure)	Robust responses (CR/PR) in heavily pretreated patients	Anemia, neutropenia, thrombocytopenia	Fatigue, diarrhea, contusion, cough

## 4 Future Directions

Future perspectives the management in the management of MCL are increasingly shaped by advances in molecular profiling and a growing understanding of resistance mechanisms. Genomic alterations such as TP53 mutations, BTK resistance mutations, and the degree of BCR signaling dependency are emerging as crucial biomarkers for therapy selection. Genomic alterations—such as TP53 mutations and varying degrees of BCR signaling dependency—are emerging as critical biomarkers for therapy selection. Among these, *TP53* aberrations are consistently associated with poor prognosis and reduced responsiveness to chemoimmunotherapy and BTKis, underlining the urgent need for alternative, chemotherapy‐free strategies in this high‐risk subset [38]. Similarly, mutations such as BTK C481S confer resistance to covalent BTKis, highlighting the importance of genetic testing before and during treatment to inform optimal sequencing and guide therapeutic decisions.

Innovations in liquid biopsy technologies, especially circulating tumor DNA (ctDNA) and minimal residual disease (MRD) assessment using next‐generation sequencing (NGS), offer powerful tools for non‐invasive, dynamic disease monitoring. These methods can detect molecular relapse before clinical progression, allowing timely intervention and potentially improving outcomes. MRD negativity is also being explored as a surrogate endpoint in clinical trials, with the potential to inform decisions on therapy duration and intensification [39, 40].

To overcome BTKis resistance and enhance therapeutic precision, novel agents such as non‐covalent BTKis (e.g., nemtabrutinib and vecabrutinib) are under clinical investigation. These molecules bind reversibly to BTK and retain efficacy against C481‐mutated forms, offering new options for patients who relapse after treatment with covalent BTKis. In parallel, BTK degraders—agents designed to induce proteasomal degradation of BTK—and dual kinase inhibitors targeting BTK alongside complementary pathways such as PI3K or SYK are in preclinical and early clinical development, aiming to achieve more complete blockade of oncogenic BCR signaling [41, 42].

Combination strategies are another major focus of current clinical research. Trials are evaluating BTK inhibitors in conjunction with BCL‐2 inhibitors (e.g., venetoclax) to harness synergistic pro‐apoptotic effects and deepen therapeutic responses [43]. Moreover, regimens incorporating anti‐CD20 monoclonal antibodies, bispecific T‐cell engagers (e.g., CD20/CD3 bispecific), and CAR‐T cell therapies aim to enhance immune‐mediated eradication of malignant clones. These multi‐agent strategies are designed not only to achieve a more durable response but also to delay or prevent the emergence of resistance [44].

In summary, the future treatment landscape of MCL is evolving toward a more personalized, biomarker‐driven, and immune‐based paradigm. Integration of molecular diagnostics with next‐generation targeted therapies and immunotherapeutic platforms holds the promise of optimizing outcomes across heterogeneous patient populations and overcoming the current limitations of therapy.

## 5 Conclusions

The therapeutic evolution of MCL exemplifies the broader shift in oncology toward precision medicine. Historically treated with intensive chemoimmunotherapy and ASCT, MCL has seen significant advances with the advent of BTKis, which have reshaped both the R/R and frontline settings. Ibrutinib, the first‐in‐class BTKi, set a new standard by demonstrating durable responses as monotherapy. Yet, its non‐selective kinase profile is associated with cardiovascular and bleeding toxicities, and resistance—most notably via BTK C481 mutations—inevitably emerges in many patients [1, 3].

Second‐generation covalent BTKis such as acalabrutinib and zanubrutinib improve on selectivity and tolerability, and now represent alternatives for those patients who are intolerant to ibrutinib. However, it is the emergence of next‐generation, reversible BTK inhibitors like pirtobrutinib that may offer the most promising path forward. These agents retain activity against C481‐mutated BTK and exhibit favorable safety profiles, even in heavily pretreated populations [14].

The TME remains an underappreciated barrier to long‐term disease control, contributing to BTKi resistance through stromal adhesion, chemokine signaling, and immune suppression. Future strategies must go beyond single‐agent BTK inhibition and address the complex interplay between malignant cells and their supportive niches. Ongoing trials evaluating BTKis in combination with BCL‐2 inhibitors (e.g., venetoclax), monoclonal antibodies, bispecific T‐cell engagers, and CAR T‐cell therapies aim to achieve deeper, more durable responses by dismantling these resistance networks [43].

Equally important is the integration of molecular diagnostics—including TP53 and BTK mutation status—and non‐invasive monitoring tools such as ctDNA and MRD assessment. These technologies are rapidly becoming essential for guiding therapeutic decisions, tailoring treatment intensity, and anticipating relapse [39].

In conclusion, BTK inhibitors will continue to play a central role in MCL therapy. However, their optimal use will depend on patient‐specific factors, genetic profiling, resistance patterns, and rational combination strategies [17]. The future of MCL management lies in a biomarker‐driven, immune‐integrated, and resistance‐adaptive approach that maximizes response while minimizing toxicity. As the treatment landscape evolves, the goal is not only to prolong survival but to achieve durable remissions with minimal burden—offering patients a more precise and personalized therapeutic journey.

## Author Contributions

Conceptualization: Santino Caserta, Enrica Antonia Martino, and Massimo Gentile. Methodology: Ernesto Vigna, Antonella Bruzzese, and Fortunato Morabito. Writing – original draft preparation: Santino Caserta. Writing – review and editing: Massimo Gentile and Fortunato Morabito. All authors have read and agreed to the published version of the manuscript.

## Ethics Statement

The authors have nothing to report.

## Conflicts of Interest

The authors declare no conflicts of interest.

## Data Availability

Data sharing not applicable to this article as no datasets were generated or analysed during the current study.
